# A transient benign lymph node-based proliferation of T-cells simulating non-Hodgkin lymphoma in a patient with psoriasis treated with tumor necrosis factor alpha and CD11a antagonists

**DOI:** 10.1186/1746-1596-3-13

**Published:** 2008-03-26

**Authors:** M Yadira Hurley, Mary Noel George, Craig L Leonardi, John L Frater

**Affiliations:** 1 Departments of Dermatology (Hurley, George, Leonardi) and Pathology (Frater), Saint Louis University School of Medicine, St Louis, MO, USA

## Abstract

**Background:**

Therapeutic biologic agents are uncommonly associated with lymphoma.

**Case presentation:**

We report a patient with psoriasis treated with the biologic agents efalizumab (Raptiva^®^) and etanercept (Enbrel^®^), who developed painless lymphadenopathy with peripheral lymphocytosis during treatment, simulating a non-Hodgkin lymphoma clinically and pathologically. Lymphocytosis and lymphadenopathy spontaneously remitted following cessation of etanercept therapy and have not recurred.

**Conclusion:**

Distinction between clinically benign lymphoid proliferations related to antipsoriasis therapy and malignant lymphoma avoids the unnecessary use of anti-lymphoma chemotherapy.

## Background

In the epidermal milieu of psoriasis, activated CD4+ helper T-cells interact with CD8+ suppressor T-cells, dendritic cells, and keratinocytes, resulting in production of T_H_1-associated cytokines, the most important of which is tumor necrosis factor (TNF) [1]. In an effort to rationally treat psoriasis, several biologic therapies have been engineered. One of these is etanercept (Enbrel^®^), a dimeric fusion protein that blocks the effects of TNFα [2]. Another is efalizumab (Raptiva^®^), a humanized monoclonal antibody that binds to the CD11a subunit of lymphocyte function-associated antigen-1 (LFA-1) to inhibit activation, trafficking to the dermis and epidermis, and reactivation of pathogenic T-cells. Use of biologics has been associated with adverse effects such as infection and autoantibody generation. There may also be a slight increase in the risk of lymphoma, but the data is controversial and may be confounded by the increased incidence of lymphoma in the general psoriasis population [2].

Like many diseases, psoriasis is postulated to induce relative immunosuppression. The increased incidence of infection and autoantibody generation suggests that further aberrations of immune status may be engendered by biologic therapy. This immunomodulation has been associated with a spectrum of lymphoproliferative disorders, some of which are clearly represent malignant lymphomas, others which are due to a dysregulated immune response. The distinction between these two extremes has important implications for selection of appropriate therapy. We report a clinically benign proliferation of T-cells simulating a non-Hodgkin lymphoma arising in a psoriasis patient after beginning treated with efalizumab and etanercept, discuss the pathologic features and choice of treatment, and review the literature.

## Case presentation

**Figure 1:**
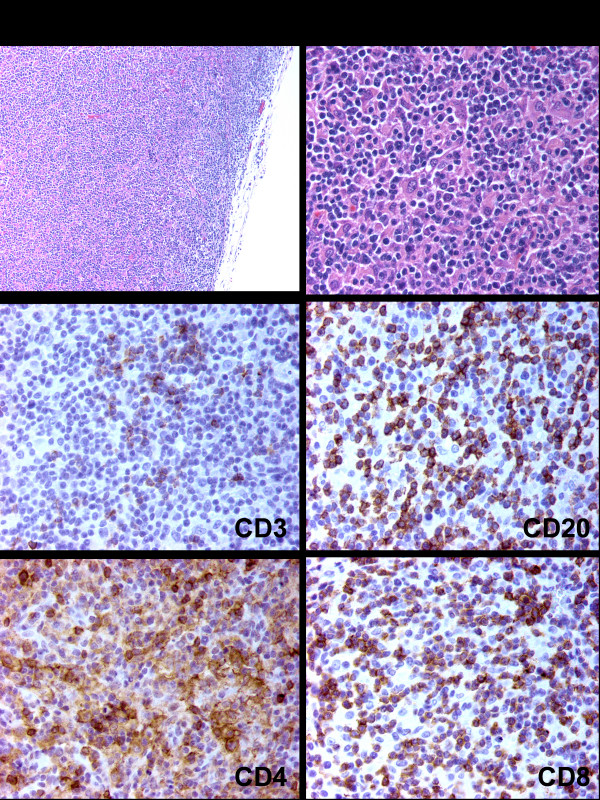
**Lymph node biopsy**. Biopsy of the enlarged lymph node revealed an intact capsule and obliterated sinuses. A diffuse infiltrate effaced the normal nodal architecture (Upper left panel, hematoxylin and eosin, original magnification ×100). The infiltrate was composed of an admixture of small lymphocytes, macrophages, and plasma cells (Upper right panel, hematoxylin and eosin, original magnification ×400). The infiltrate was composed of a mixture of CD3 positive T-cells (including both CD4 and CD8 positive cells) and CD20 positive B-cells. Numerous macrophages were also CD4 positive (Original magnification ×400).

**Figure 2:**
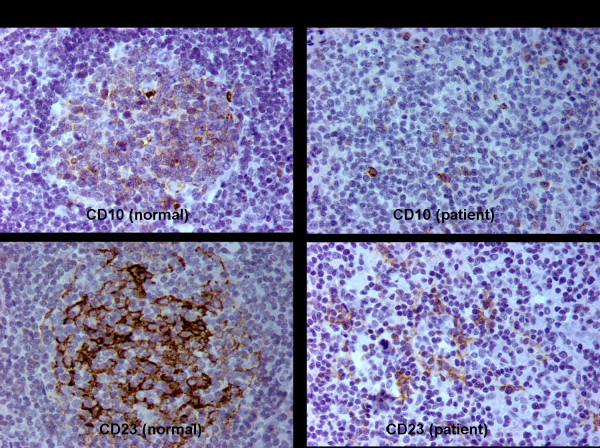
**Lymph node biopsy**. Follicular destruction was demonstrated with immunohistochemistry, which revealed small clusters of CD10 positive small lymphocytes, corresponding to the residual germinal centers (Upper right panel, original magnification ×400). CD23 immunohistochemistry showed the dendritic cell network to be essentially obliterated (Lower right panel, original magnification ×400). For comparison, normal germinal centers stained for CD10 and CD23 are illustrated in the upper left and lower left panels, respectively (Original magnification ×400).

A 36 year-old white woman with an 11-year history of psoriasis who after having maintained relatively good control with class I topical steroids and calcipotriene ointment flared to 30% total body surface area involvement. She was unresponsive to a 9 month course of efalizumab (Raptiva^®^) 1 mg/kg/wk including a 5 month overlap with methotrexate at 5 mg/wk. She was then transitioned to etanercept (Enbrel^®^), 50 mg subcutaneously biweekly: efalizumab was overlapped 4 weeks to avoid psoriasis worsening. Psoriasis worsening has been reported when patients discontinue efalizumab, in particular patients that that are partial or nonresponders. Over the course of these overlap weeks she developed non-tender, bilateral cervical lymphadenopathy. She denied other symptoms. A complete blood count performed at this time revealed a white blood cell count of 11.9 k/mcl, hemoglobin 14.6 g/dL, and platelet count of 218.0 k/mcl., and a differential count included 30.8% neutrophils, 62.2% lymphocytes, and 7.0% monocytes. Circulating lymphocytes were small and mature-appearing. A lymph node biopsy was performed. Microscopic examination of the enlarged lymph node revealed diffuse effacement of the normal nodal architecture by an infiltrate composed of small lymphocytes, macrophages, and plasma cells. The capsule was intact and thin and the nodal sinuses were obliterated. The lymphocytes were a mix of CD4 positive T-helper and CD8 positive T-suppressor cells. Although follicles were not apparent in sections stained with hematoxylin and eosin, immunohistochemistry revealed small clusters of CD10 and BCL6 positive small lymphocytes, corresponding to residual germinal centers. CD23 immunohistochemistry showed the dendritic cell network to be essentially obliterated (Figures 1 and 2). Plasma cells were present individually and in variably sized clusters located throughout the node. By immunohistochemistry, they were composed of a polyclonal mixture of kappa and lambda light chain positive cells. Flow cytometric analysis of the peripheral blood revealed a lymphocytosis composed of a mixture of CD4+ T-helper and CD8+ T-suppressor cells. The patient was instructed to stop all biologic agents. The lymphadenopathy resolved over the following 2 months, and the patient remained without adenopathy for 2 months. At that time etanercept was restarted at 50 mg subcutaneously weekly with good control of her psoriasis and no recurrence of her adenopathy for 23 months.

## Discussion

We report a self-limited nodal-based clinically benign proliferation of T-cells developing in a patient with psoriasis treated concurrently with the TNFα inhibitor etanercept and the anti-CD11a antibody efalizumab. To avoid the unnecessary use of anti-lymphoma chemotherapy, it is important to recognize that these entities do not behave in a malignant fashion. Our case is difficult to categorize on the basis of the histopathologic findings alone. The lymph node had several hallmarks of malignancy, including diffuse effacement of the normal nodal architecture and obliteration of the sinuses. Although a phenotypically abnormal T-cell population was not identified by flow cytometry or immunohistochemistry, this does not rule out the possibility of a peripheral T-cell lymphoma, some cases of which show normal immunophenotypes. Although PCR-based T-cell receptor gene rearrangement studies are sometimes employed in an effort to assess clonality in cases of suspected T-cell lymphomas, the presence of a clonal result is not diagnostic of malignancy, and may be seen in a variety of benign conditions. For this reason, and because the symptoms ameliorated following cessation of therapy, suggesting a benign condition, we did not perform gene rearrangement studies. The identification of other cell types besides lymphocytes in the infiltrate also does not exclude a malignant lymphoma. In fact, a heterogeneous background population is often identified in lymph nodes involved by T-lineage lymphomas. The development of lymphadenopathy after combining anti-TNF and anti-CD11a therapy and the prompt resolution of symptoms after their discontinuation, followed by lack of recurrence with using anti-TNF therapy alone, suggests that these drugs caused or facilitated the development of this process. Furthermore, the complete resolution of symptoms without the use of chemotherapy suggests that this lymphoproliferative disorder to clinically similar to disorders occurring in other immunocompromised patient populations.

Lymphoproliferative disorders occurring in the immunosuppressed/immunocompromised population share many characteristics and have been classified as "immunodeficiency associated lymphoproliferative disorders" in the World Health Organization Classification [3]. Some cases, particularly in the post-transplant setting and in methotrexate-associated lymphoproliferative disorders, show a premalignant disease state with an antigen-stimulated polyclonal proliferation of lymphocytes (usually B-cells). Distinction of this premalignant phase of disease is important, particularly in immunosuppressed patients, since modulation of the patients' drug regimen often results in resolution of the disorder without the use of chemotherapy.

Psoriasis induces a relative immune deficient state and it is likely that the resulting loss of immune surveillance results in an increased incidence of lymphoma in this population [4,5]. This tendency may be exacerbated by the use of immunosuppressant and immunomodulatory drugs such as methotrexate and the new biologic medications that further dysregulate the immune system.

Since the approval of TNFα inhibitors, there have been several reports of non-Hodgkin lymphoma arising in this background. The largest series included 18 patients treated with etanercept [6]. Notably, two of these individuals had a history of psoriatic arthritis; their specific characteristics could not be extracted from the other cases due to the presentation of clinical information. Besides the above study of Brown et al, there is currently minimal other data regarding the risk of lymphoma in psoriatic patients. Adams et al reported a fatal aggressive cutaneous T-cell lymphoma developing 18 months after initiation of etanercept for psoriatic arthritis [7]. Although less frequent, Hodgkin's and non-Hodgkin's lymphoma have also been reported in patients treated with efalizumab [8].

However, some lymphoid proliferations occurring in patients treated with biologics may be successfully treated without the use of antineoplastic regimens. T cell development and recruitment are facilitated by numerous cytokines including TNFα, which acts as a potent mediator of cell death in cells expressing its receptor [9]. In this capacity TNFα has important functions in inflammation and host antitumoral responses. TNFα also interacts with a variety of other transiently expressed ligands such as CD30 and CD95 (FAS), which are expressed on numerous cell types including activated T cells [9]. Thus, blocking TNFα would be expected to result in a dysregulation of these highly choreographed interactions, resulting in a non-malignant expansion of cells expressing TNF ligands, including activated T cells. Furthermore, when coupled with an anti-CD11a antibody these activated T-cells are excluded from the skin resulting in compartmentalization in the blood and likely lymph nodes. In fact, treatment with efalizumab results in a mean increase of white blood cell count, a doubling of mean lymphocyte counts and an increase in eosinophils counts due to decreased leukocyte adhesion to blood vessel walls and decreased trafficking from the vascular compartment to tissues. We postulate that these mechanisms are responsible for this patient's lymphoid proliferation. Therefore, during transition periods when overlapping these biologic therapies, clinicians should be aware of the potentiating side effects, which could result in transient lymphadenopathy simulating a malignant lymphoproliferative disorder.
